# Supplementary material for: Deletion of the SELENOP gene leads to CNS atrophy with cerebellar ataxia in dogs
Source: PLoS Genet. 2021 Aug 2;17(8):e1009716. doi: 10.1371/journal.pgen.1009716 (PMC8360551; doi:10.1371/journal.pgen.1009716)
Supplement: S1 Fig — (PDF) [file pgen.1009716.s002.pdf]

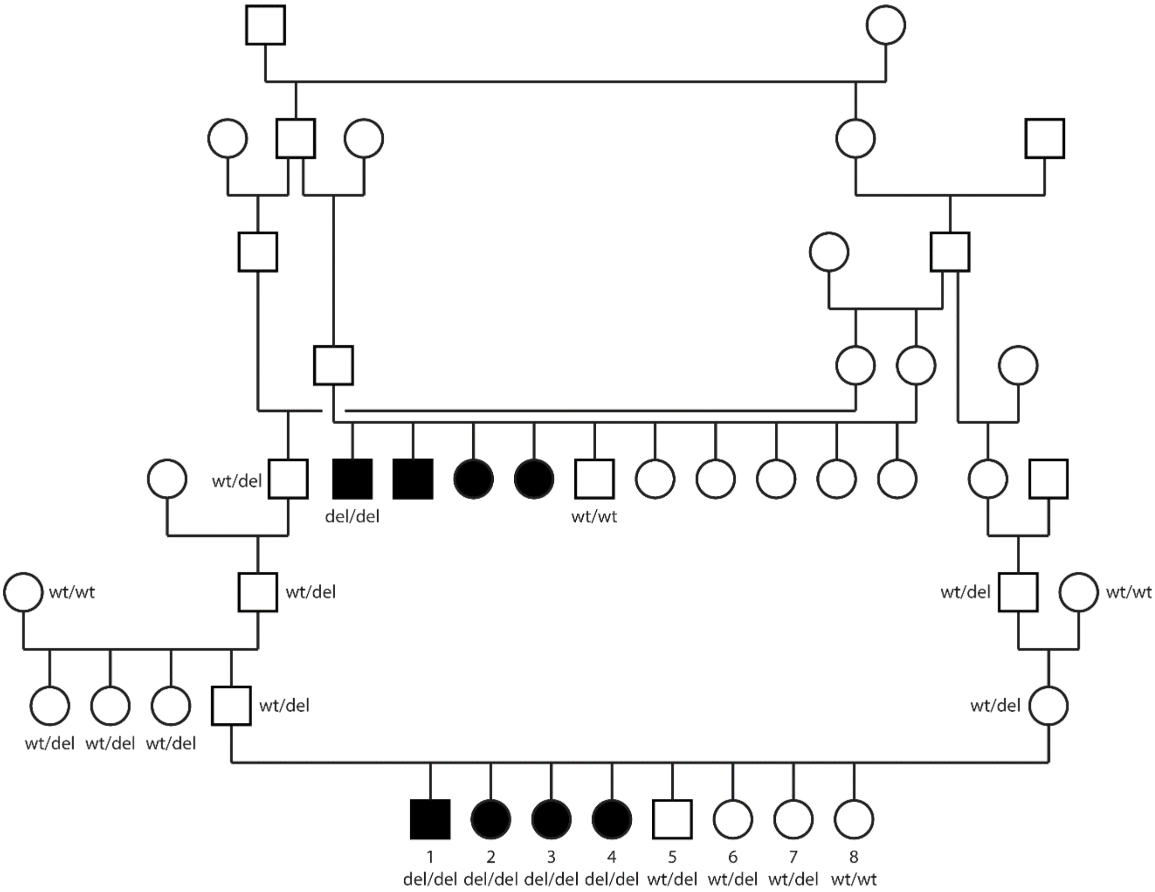

S1 Figure. Pedigree of Belgian Shepherd dogs with ataxia. Affected dogs are indicated with filled symbols. The index family with 4 affected puppies is shown at the bottom of the pedigree. Genotypes at the *SELENOP* deletion (Chr4:66,946,539_66,963,863del17,325) are indicated for all dogs, from which a DNA sample was available. During the investigation, we discovered one additional dog homozygous for the *SELENOP* deletion that also showed ataxia. This dog was born in a litter of 10 puppies shown in the middle of the pedigree. The three other reportedly affected puppies from this litter were euthanized as young puppies and no samples for genetic analyses were available. Typical for purebred dogs, all affected dogs are inbred and share common ancestors on the paternal and maternal side. The pedigree is suggestive for a monogenic autosomal inheritance of the trait.
