# Supplementary material for: Deletion of the SELENOP gene leads to CNS atrophy with cerebellar ataxia in dogs
Source: PLoS Genet. 2021 Aug 2;17(8):e1009716. doi: 10.1371/journal.pgen.1009716 (PMC8360551; doi:10.1371/journal.pgen.1009716)
Supplement: Attachment — Submitted filename: 2021Jul06_Cover_letter_R1.pdf [file pgen.1009716.s009.pdf]

b
UNIVERSITÄT
BERN

Institute of Genetics
Vetsuisse Faculty

Director

Institut für Genetik, Bremgartenstr. 109a, CH-3001 Bern

Editor
PLoS Genetics

Bern, 06-July-2021

Revised manuscript for publication in *PLoS Genetics*, PGENETICS-D-21-00621

Dear Prof. Dr. Tiret, dear Prof. Dr. Williams,

Thank you very much for the positive evaluation of our manuscript entitled **„Deletion of the *SELENOP* gene leads to CNS atrophy with cerebellar ataxia (CACA) in dogs"**. Please find enclosed our revised manuscript. With respect to the comments we made the following changes:

Reviewer 1:

(1)
Fig 1. The figure is not very illustrative. The authors should consider to exchange the figure with videos if possible/available.
Response: We added two supplementary videos that should illustrate the clinical phenotype much better than the still photos in Figure 1. We would like to keep Figure 1 in the main text as this figure may still be useful to the superficial reader who does not take the time to view the supplementary data.

(2)
Under "Necropsy and histopathological examination"- the authors have not explained what is used as a healthy control (breed/age)? Please add.
Response: We added breed and weight of both control animals to the Materials & Methods section.

(3)
Fig 3. The figure contains a lot of information but is a suboptimal. The primers F1/R1 and R2 could be moved/lifted closer to the IGV-sequence. The graphical presentation/naming of the three CCDC152 could be improved.

Prof. Dr. Tosso Leeb
Institut für Genetik
Bremgartenstr. 109a, Postfach
CH-3001 Bern

Tel +41 31 631 23 26
Fax +41 31 631 26 40
Tosso.Leeb@vetsuisse.unibe.ch
www.genetics.unibe.ch

Response: We modified the figure according to the suggestions of the reviewer. Unfortunately, it is not possible to explicitly label the three different *CCDC152* isoforms in a legible font size due to space constraints. We also revised the figure legend and hope that the given information is now easier to grasp.

(4)
Selenium measurements was performed in frozen blood samples of the eight puppies and shows a significant reduction in affected and heterozygotes. It is mentioned that SELENOP "is required for the transport of selenium into the brain and other organs". How well is blood concentration of selenium in this case a good expression for the tissue content? Does "required" mean that it is expected that the concentration in brain is "close to zero" when the gene is deleted (or do the authors mean "involved in transport of selenium")? The clinical variation is interesting, but maybe a variation in selenium-supply to puppies would not be big within the same litter?
The authors should shortly discuss the value of blood selenium in the situation where the involved gene has a role of transport into CNS, and why selenium was not measured in brain (lack of appropriate tissue-samples?). Also, if there is any knowledge about correlation between blood and tissue concentration of selenium in knockout mice? Could other genes be responsible for the clinical variation (like the mentioned LRP8 and Trsp) or do the authors believe that selenium supply is the only variable? If such information is missing the value of spontaneous models might increase.
Response: We appreciate this valuable comment by the reviewer. We added a sentence to the discussion stating the strong correlation between blood and brain selenium levels in *Selenop*$^{-/-}$ knockout mice ("Both blood and brain selenium levels were decreased by approximately 70%-80% in *Selenop*$^{-/-}$ mice [15]."). These results indicate that selenoprotein P is probably not the only, but by the far the most important means of selenium transport to the brain. We therefore did not change our wording that selenoprotein P is "required" for selenium transport into the brain.
We indeed do not have the appropriate samples to perform selenium measurements in brain of the affected puppies. Please keep in mind that these were clinical ataxia cases that were euthanized shortly after presentation. It took several months before we elucidated the causative genetic defect and at that time, unfortunately, no further samples could be obtained. The proposed selenium measurements in brain (or in specific brain regions) are definitively planned for the future, if further affected puppies should become available to research.
Finally, we proposed different levels of dietary selenium intake as one possible explanation for the observed variability in the severity of the clinical phenotype in the affected dogs (mainly between the four puppies, which were euthanized at 4 weeks of age and the fifth affected dog, which reached an age of ten years). There are certainly many more possible alternative explanations, such as genetic variation mentioned by the reviewer. As we don't have experimental data on this, we refrained from adding a long and very speculative discussion.

UNIVERSITÄT BERN

Reviewer 2:

(1)
The paper by Christen et al. describes the natural deletion of SELENOP in dogs as a potential new model of studying Se-metabolism. The topic and the study design facilitates the publication of this manuscript with the current journal, in my opinion. The paper uses adequate methodology and is well written regarding language, style and word usage. I would recommend a minor revision, however, a section of the study limitation is currently missing so I have to recommend a major revision at this point. Please, disclose the limitation of your study (and strengths if you wish so too) in a separate section or just as a part of your discussion. One of the limitation is probably unknown Se intake for the progenitor dogs and offspring as an example. That is my only major comment.
Response: We thank the reviewer for the kind comments. We added a paragraph to the discussion outlining some limitations of the study.

(2)
I would recommend omitting CACA abbreviation from the title, you have it in the abstract for indexing purposes already.
Response: Revised accordingly.

(3)
Running title is irrelevant, I would say "SELENOP deletion leads to cerebellar ataxia in dogs" highlights the content of the paper much better (still <70 characters).
Response: Revised accordingly.

(4)
Necropsy and histopathological examination section: State here which tissues were examined in brief, refer to Material section if needed to avoid major repeats. Otherwise, it is hard to follow. In line with that, testes are severely damaged in Selenop-/- mice leading to male infertility even under Se-rich diets (see studies by Schomburg's group at Charité and other groups). Was it investigated in SELENOP deleted dogs? Any plans to go into that later, if not?
Response: We added all examined tissues to the Materials & Methods section. Unfortunately, testes of the only male affected puppy were not collected and therefore could not be examined histologically. This would indeed have been very interesting and the collection of testes from future CACA cases in Malinois puppies is needed to address the question.

(5)
Discussion:
a) the second sentence: Mention here which chromosome contains *SELENOP* gene in dogs as a service to the reader.

Response: Revised accordingly.

b) the third sentence: To be accurate, this is true for vertebrates but not generally true.
Response: We added “in vertebrates” to the general statement.

c) "To the best of our knowledge, there are no reports of human patients with genetic variants in *SELENOP* or other spontaneous *SELENOP* mutants in any mammalian species", I think SNPs of *SELENOP* gene in humans may be worth mentioning in this section. There were a few relevant studies about that (e.g. by Méplan's group). Additionally, Bellinger's group hypothesised that SELENOP might have some signalling functions in the brain through LRP8 (doi: 10.3233/JPD-2012-11052), which may be partially responsible for the neurological phenotype observed.
Response: We modified the sentence in order to make it clear that there are no reports of human patients with neurological dysfunctions caused by *SELENOP* variants. To the best of our knowledge, the association studies by the group of Catherine Méplan investigated a possible association to cancer, but not to early-onset neurodegeneration. We added a sentence and two additional references mentioning the hypothesized role of selenoprotein P in brain signaling.

d) Necropsy, histology, immunohistochemistry section: space missing – 2µm.
Response: Revised accordingly.

Reviewer 3:

(1)
Abstract
Is CNS (central nervous system) a commonly used abbreviation? Otherwise, please define.
Response: We think that the abbreviation CNS does is widely known and does not need a verbose explanation.

(2)
Please replace wt/wt by homozygous wildtype.
Response: Revised accordingly.

(3)
Introduction
Reference 6 (2014) could benefit to be completed by a reference to the OMIA web site (more recent update).
Response: We added a reference to the OMIA database as requested.

(4)

Results

Linkage analysis does not reach statistical significance threshold. Max LOD score < 3. Please add this information in the text.

Response: We added "with a maximum LOD score of 2.31" to the text.

(5)

The fifth affected dog that is homozygous for the deletion is related to the four affected puppies (Fig S1). Please add this relevant information in the text.

Response: Revised accordingly.

(6)

Supplementary Materials

S1Table: Please add some information:

- LOD score values for first and last SNV from the linked interval
- ID of the SNV with the maximal LOD score
- legend for the last four columns of the "Homozygosity visual Chr4" table

Response: We thank the reviewer for this valuable comment as it alerted us to a technical error in our original linkage analysis that led to the exclusion of some valid markers. We now repeated the linkage analysis with all 95,207 markers. This did not change the detected linked genome segments.

We modified S1 Table in the following way:

- We added another tab with the raw Merlin output for the linkage analysis. There were two genome segments that showed positive LOD scores: A very large segment on chromosome 4, where >2000 makers show identical LOD scores of 2.3054. At the extreme edges of this segment, less than 10 markers show smaller, but still positive LOD scores. The second linked segment on chromosome 18 comprised only 9 markers with LOD scores ranging from 1.054 to 1.4761.
- Unfortunately, the IDs of the markers are not directly visible in the Merlin output. However, as the genome position is given, we hope that this is enough information for the reader to identify each marker. The LOD scores are now given for all 95,207 markers.
- The last four columns of the "Homozygosity visual Chr4" table contained simple Excel formulas subtracting allele 1 from allele 2 for the four affected dogs ("Allele 2 – Allele 1"). This helped us to highlight genotypes that are not homozygous. However, this information is definitively not essential and may be confusing to the reader. We therefore deleted these four columns.

  The deleted columns can very easily be re-constituted from the raw data shown in columns H-O.

All changes in the manuscript are in indicated in red. If you have any further questions regarding our manuscript, please do not hesitate to contact me at any time. We are looking forward to hearing your decision whether the revised manuscript can now be published in *PLoS Genetics.*

Yours sincerely,

Prof. Dr. Tosso Leeb
